# Factors Entering in the Maintenance and Control of Free Dental Dispensaries*Presented to Eighth District Dental Society, State of New York, Dec., 1912.

**Published:** 1913-02

**Authors:** Wm. W. Belcher

**Affiliations:** Rochester


### Factors Entering In the Maintenance and Control of Free Dental Dispensaries*

By WM, W. BELCHER. D.D.S.,
Rochester
Editor of *Dental Dispensary Record*

ASSUMING a free dental dispensary as essential to the well being of those sadly needing services and unable to pay, under whose auspices should it be established and by whom controlled?

Shall it be that of a private charity, the local Society for the Prevention of Cruelty to Children, the Society for the Prevention and Control of Tuberculosis, the Health Association or the local Health Officer?

Under these conditions, the dentist is called in and his services paid for and the charitable organization determines the worthiness of all applicants.

Shall the local School Board establish the free dental dispensary as a part of its fight in securing a whole body and a whole child as a teaching factor? In this case, the work is oftimes associated with the eye and ear, the nose and throat clinic with an economy of operation. The teacher or school nurse determines the worthiness of applicants. The dental operator is asked to supply the services and when the worthiness of the work is demonstrated, the funds for maintenance is added to the school budget and is a part of the school work.

Shall the free dental dispensary be established by the municipality or supported in part by municipal moneys? This is really where the burden belongs and the plan of the work in Germany, in a large degree, and it is here the dental dispensary has been most successful and longest established.

Atlanta, Ga., is about to try a new plan—new, at least, to the essayist. The local Board of Trade have asked the dentists of the city to establish a free dental dispensary for the worthy poor school children. This insures continued financial support and a maintained interest from the best citizens of the community.

Finally, shall the dentists themselves establish their own dispensary; receiving full credit for the work and determine the worthiness of all applicants as best they can? Shall they finance and manage their own charity?

To do this it will be necessary to call on the public for support. The Dental Society must supply enough volunteer operators or employ a paid operator, and each pledge himself for the amount necessary to reimburse the dental interne and the dental nurse. Then there are supplies and other essentials that will average from 15 to 20 per cent. of the total expenses. If the local dentists are to finance and manage their own charity, where

* Presented to Eighth District Dental Society, State of New York, Dec., 1912.

are the funds to come from? Who is to manage these dispensaries? Every day new problems present themselves. They must be decided at once. Who is to do this? By what means and methods are you to determine the worthy from the unworthy, that your charity shall not be a reproach to your society and the community? Gentlemen, these are important questions and not to be lightly passed on. On their wise decision depends in a large degree the success or failure of the work.

Every plan enumerated has its good points and its weak ones. Some of these I will hurriedly touch on and so not tire you with a mass of detail. When a man gets interested in a subject, he is apt to confuse his hearers with a mass of detail, all important to his mind and essential in the proper presentation of his subject.

A specialist in the study of the wagon wheel could write a book on the subject. Who invented it? Nobody knows. The wagon wheel along with the dog and the family cat have been with us since we have any record of man. But the hub with its bearings of steel balls or hardened steel rolls, and again, the spoke of wood or wire, of such and such a dimension, of such a number and tensile strength. And the tire, who invented the tire? Of wood, of leather, of steel, of rubber. Solid rubber at first and then with its cushion of air, with its inner tube and without. And then the rim. Removable rims of different types. What a discussion presents itself to even the untrained mind. Of course the specialist could write a book on the subject.

To avoid these errors, I will have to confine my remarks to two aspects of the subject of free dental dispensaries and regret that it should not be only one.

Let us take up first the municipal establishment and control. This is the goal we are all striving for, and at first glance seems the most desirable; under proper restrictions I think this is true, but, alas! there is a fly in the ointment. After having established a free dental dispensary and educated the public and school authorities as to the necessity and desirability of the work of caring for the teeth of the worthy poor who are unable to pay, it would almost seem our troubles had ended, but, unless careful planning has been done, they have only begun.

Let me introduce to you this colored gentleman in the wood pile. Carefully remove each stick. He is a very elusive mortal and not so easily located. His name is Ebenezer Erastus Politics. Yes, here he is; we have successfully uncovered him. Let us examine the rascal at our leisure and in daylight. He is a likeable fellow. He is acquainted with most of your friends. He has done you many a favor and stands ready to do you more. He has a "machine" cunningly devised and far reaching in its workings. Sly as a fox, wise as an owl, and like them both combined, he sees in the day time and in the darkness of the night.

Let me illustrate my position by the story of one of our local charities. It has been fairly well managed in the past and dependent on the public for support. Using its influence with the powers that be, it succeeded in getting an appropriation of $2,000 of the municipal funds. In addition to this they had a "Flower Day" and all the political bosses, from the man "higher up" to the employee who shovels the coal, helped to boost, and the daughters and wives went on the streets of our city and accosted the men they knew and those they did not know with an appeal to purchase a flower for the support of the charity. You paid ten cents or ten dollars for a dinkey flower which the lady tucked in your buttonhole, the amount depended on your charitable spirit or the impression you wished to convey to the fair peddler of flowers. Do you approve of this means of raising money? I do not. A "Field Day" with the politicians and others figuring in ball games and various contests followed. From these combined sources the sum of $8,000 was realized.

One of our free dispensaries is housed in the same building with this organization, although it is entirely separate, and none of the funds are used to support the dental charity, other than the furnishing of a room with heat and light, the same courtesy we receive from the public schools where are located two of our free dispensaries.

Comes the director of this organization to the dentist in charge of the Dental Dispensary and says: "This child is sent to have her teeth attended to by Commissioner Blank. I wish you would fill her teeth without investigating the case as Commissioner Blank is a good friend and supporter of our work." The politician who has played baseball, who has allowed his daughter and his wife to go out and raise money for the organization, has a washwoman whose children's eyes need attention. "Can she get relief at the dispensary?" Sure thing. I'll write you a note and it will be O. K. The director knows me and the kid will be attended to P. D. Q." The washwoman has a piano in the front parlor. Her husband is employed and their home is paid for. She is a taxpayer. Does the director investigate? Nix. That charity has gone to the bad. It isn't a charity any more. This matter of paying taxes is considered a privilege for aid at any municipal supported charity. We have had this argument used by a woman with an only child bedecked with fine linen and fancy hair ribbons. Her daughter was refused treatment. She waxed wroth. She had a right to have her daughter's teeth treated. Wasn't she a taxpayer! She was told that none of her tax money supported the dental dispensaries, and the fact of her being a taxpayer was one of the strongest arguments she could offer against the free treatment of her child.

These conditions are not a fairy story, it is a factor in every dispensary receiving municipal support that I am acquainted with. What is the result? The reaction is coming and the city fathers, learning of these things are about to curtail the appropriations for support. They, themselves, or part of them, may have been guilty of the acts they condemn in others, but that does not help matters.

Here is a part of a letter recently received from a prominent practitioner who is interested in the free dental dispensaries of his city which is receiving municipal support: "I was called on to-day by a very influential Councilman, who told me there has developed a good deal of opposition to our free dental clinic. The complaint is that too many children are treated there whose parents could well afford to pay for regular dental services. In this way too few of the worthy poor receive attention.

"How do you manage in other cities to keep out the well-to-do? You probably have some plan and I should like to learn of it. The annual city appropriation here is over $9,000, and there is talk of cutting this amount materially. We must prevent it if we can. Give us some help and promptly so as to save the day."

Now, I don't know the local cause of this upheaval, but I can guess, and unless part of the money appropriated is used to employ some one with a stiff back bone and absolutely indifferent to personal influence, who is of good judgment and both sane and sensible, with funds for special investigations, the Dental Dispensary in this city had better close its doors as a *charity*.

I do not blame these gentlemen who expect favors. It is only human nature. I consider our city of Rochester a well-governed city with an absence of any big grafting schemes. I have never met our local boss: "Uncle George Aldrich," but I have a sneaking liking for him, and all the politicians I know are royal good fellows. Personally, I have a lively appreciation of favors extended me and if I were in politics I am afraid I would be a very weak sister when it came to refusing favors to a man who had befriended me. But it isn't a matter of men; it's a matter of principle. A political "charity" is an impossibility. They won't mix any more than oil and water.

Passing for a time the municipal dispensary we will consider one under the control and supervision of the dental profession. If you will pardon the personal part, I know of no place where this is done in its entirety as at Rochester, N. Y.

In the beginning, this work was supported by the gifts of our patron, the late Capt. Henry Lomb, and the equipment in our three dispensaries has been very largely donated. For the past five years it has been supported entirely by voluntary contributions from the public, our Rochester Dental Society and the production of two theatrical entertainments. The production of these has been somewhat strenuous and it has been questioned if the same effort expended in other directions might not be more productive. Perhaps this is true, but the character of these entertainments have been of such a high order and the advertising of our work so effective that we look upon the direct proceeds as only part of the compensation. The added publicity through the press is perhaps worth all the effort expended, outside of any financial gain.

The management of the work in Rochester is in the hands of the Board of Directors, seven in number and one honorary member, the essayist. This board attend to all the routine business of the society and looks after the free dental dispensaries. They elect their own chairman and appoint a dispensary committee, one man to each dispensary. He is held responsible for the dispensary under his control. All requisitions for supplies are made out to him. He furnishes the reports and is supposed to make frequent trips to the dispensary to see that everything is working smoothly. The board elects its own secretary, who keeps the minutes of the meetings, and also a treasurer, who is under bond and has the keeping of the dispensary funds. He makes out a report every two months of the sums paid out and the balance in his hands. Thus, the dispensary funds are kept entirely separate from those of the society. The DENTAL DISPENSARY RECORD is published by the society, but it has an entirely separate organization and its own funds and treasurer. Each part of our organization is kept separate and stands or falls on its own merits.

Now comes the question, "How do we guard against unworthy applicants?" In the first place, we are chartered and under the supervision of the New York State Board of Charities. Each applicant makes out a card as to their family history and the total income. This is sworn to and they are issued a card with a penalty clause on the back reading as follows:

PENALTY FOR FALSE REPRESENTATIONS.

Section 25, Chapter 368, Laws of 1899.

Any person who obtains medical or surgical treatment on false representations from any dispensary licensed under the provisions of this act, shall be guilty of a misdemeanor, and on conviction thereof shall be punished by a fine of not less than ten dollars and not more than two hundred and fifty dollars.

(Imprisonment until fine is paid may be imposed. Code Crim. Pro. 718.)

In conducting a charity you must have some rule as to the total family income. We have placed this at $2.00 per member. Thus, if a family of mother and father and three children apply for services and their income is $10 per week they are admitted if the investigation shows they have told the truth. This is rather a meager income and it has been said that it favors the foreign born population rather than the American born. This rule is not an arbitrary one. If the family have sickness; if they are handicapped by debt or the father is temporarily out of work or a dozen other reasons, they are admitted if, in the judgment of those in charge, the case is a worthy one. With us it has not been always who was worthy but who was most so. We have been driven with applications from children who sadly needed attention and the problem has been at times to select the most worthy.

How do we determine the worthiness? We call up the employer, the family physician. Best of all, we have the United Charities Association. This is a Rochester institution and is a clearing house of information for those who have applications for charity. They have an office and a trained force compiling information. In addition to this, they have correspondents in over 200 cities of the United States. Over 40,000 names of people asking charity are on the books of the association. You call up the central office and they give you the names, one, two, three—perhaps a dozen charity organizations who have been in touch with the family, and the report of their investigator is given you. It may be any one of forty charity organizations who are reporting each case that comes to them for aid or relief. We make these investigations ourselves. The school dental nurse has funds to use for the payment of car fares, etc., in making investigations. Then there is the school nurse and the teachers who are in close touch with the family, but even they are deceived occasionally. A Polish or Italian family are in apparent destitute circumstances. They live in two rooms and take in a lodger. Children everywhere. They have no rubbers, so must stay at home; no shoes, so must remain home; no school books. The kind-hearted teachers see that their wants are supplied. The first thing you know this destitute family have purchased a row of houses or a hotel. The paterfamilias has a roll of money that would choke a horse. This is a free country and everything in it is free to these people, and if they can fool you and secure charity it is considered a mark of cunning, and the only disgrace is in being found out. Yes, we have these cases and some others, and we don't know how to eliminate them, but the children have been benefited even if they have fooled you. They didn't come to the dentist until they sorely needed the services and would rather break into jail than try it again.

Shall a charge be made for services rendered? At Newark, N. J., they have tried making a nominal charge for each filling, but it has not worked out satisfactorily; some can pay absolutely nothing. The practice abroad is mostly free services to the worthy poor. About one-quarter of the German dispensaries make a small charge. In some cases it is .05 mark per filling, in others a charge of from 1 to 2 marks are made for annual treatment. Thus a child pays either 25 cents or 50 cents and receives a card good for a year's treatment at the Free Dental Dispensary. A charge of 50 cents would pay a large part of the running expenses of a dispensary once it was established.

In Rochester it has cost for the past three years from 59 cents to 69 cents for each child; this covers all expenses, including postage and printing. A charge of 50 cents would go a long ways in meeting these expenses, and I am inclined to think that no family is too poor to meet this small fee, and if they could not, then some one interested in the family would furnish the wherewith. Indeed, funds for these cases would be donated. It is a rare thing for any one to value services for more than is charged, and among the ignorant poor, particularly the foreign element, I think the effect would be good. We have had no practical experience in this matter and my views are entirely theoretical. We have been taking care of the teeth of the worthy poor for over seven years, and it will be our eighth anniversary in February. Municipal support that we can accept seems as far off as when we entered upon the work. The public are intensely interested. We have loyal friends and supporters and the time will come when the work will be taken up by the city or the school board and carried on under our supervision and control.

In Genesis, xxix chapter, xx verse, we read: "And Jacob served seven years for Rachel; and they seemed unto him but a few days, for the love he had to her." The paterfamilias must have liked his brand of work and wanted to retain his services. He sold the unsuspecting Jacob a lemon in the shape of his elder daughter, Leah, and Jacob, so it is said, didn't know the diffrence till the morning. So he served seven years more and at last Rachel was given to him. It's dollars to doughnuts Jacob made sure long before dawn, that he had not been made the victim of a cheap political trick a second time.

We have served, like Jacob, seven years, and are on the second lap of seven more; we want municipal support, but if it has to come through politics then I, for one, will be willing to see our charity work go down and out. God knows how hard we have worked. Some of us have neglected our business and given the best that was in us to make it a success. Rather abandon it than it a political prostitute posing as charity.

---

TREATMENT OF SHOCK.—Edgar A. Vander Veer and J. L. Bendell, Albany (*Journal of Obstetrics*, October, 1912). These authors (contemporaneous writers in other journals ditto) deprecate wild and indiscriminate use of drugs for post-operative collapse and shock. They, in common with others, agree that the stimulant armamentarium of the surgeon should be: 1. Saline infusion; 2. Morphine; 3. Camphor oil; 4. Adrenalin, of importance in the order maintained. Other drugs, as strychnine, atrophine, digitalis, nitroglycerine, bandy and musk are of value notwithstanding.

